# Experimental Study of the Discharging Process of Sorption Heat Storage Units Filled with 13X Zeolite

**DOI:** 10.3390/ma18235327

**Published:** 2025-11-26

**Authors:** Beata Pytlik, Daniel Smykowski, Piotr Szulc, Tomasz Tietze, Beata Anwajler, Artur Chorążyczewski

**Affiliations:** 1 Department of Energy Conversion Engineering, Faculty of Mechanical and Power Engineering, Wrocław University of Science and Technology, Wybrzeże Wyspiańskiego 27, 50-370 Wroclaw, Poland; beata.pytlik@pwr.edu.pl (B.P.); daniel.smykowski@pwr.edu.pl (D.S.); piotr.szulc@pwr.edu.pl (P.S.); tomasz.tietze@pwr.edu.pl (T.T.); 2 Department of Control Systems and Mechatronics, Faculty of Information and Communication Technology, Wrocław University of Science and Technology, Wybrzeże Wyspiańskiego 27, 50-370 Wroclaw, Poland; artur.chorazyczewski@pwr.edu.pl

**Keywords:** sorption thermal energy storage, zeolite 13X, water vapor adsorption, heat balance modeling, thermal efficiency, neural network predictive control

## Abstract

The paper presents the experimental study of the zeolite heat storage unit discharging process in a laboratory scale. The Authors focused on the discharging process, which utilizes adsorption of water, in the form of steam, on zeolite, because the adsorption process is considered as more challenging in terms of reaction kinetics and heat transfer. The Authors designed and built a laboratory stand with a sorption heat storage unit filled with 13X zeolite and with a separated heat transfer fluid system, where air was used for discharging. Dynamic parameters including the temperature of inlet and outlet air and the temperature distribution inside the zeolite bed during the discharging process were investigated. The gathered measurement data were used to determine the heat fluxes and to compute dynamic heat balance of the thermal storage unit including internal and external heat losses. It was demonstrated that the applied design and scale of the thermal storage unit allows to reach the thermal power over 300 W and heat the discharging air from 40 °C to over 110 °C. The innovative aspect of the study is the improvement of operational stability of the sorption heat storage unit through the implementation of a heat exchanger design that separates the heat transfer fluid from the zeolite bed, as well as a control system with a neural network layer for predicting the mass flow rate of steam.

## 1. Introduction

Modern energy systems face numerous challenges related to the need to increase energy efficiency, integrate renewable energy sources (RES), and utilize waste heat. One of the key technologies serving to enhance the utilization of renewable energy sources and waste heat is Thermal Energy Storage (TES) [1,2]. The increasing prevalence of solution-seeking in this field is represented by the number of publications dedicated to heat storage, which increased from 969 articles in 2015 to 2539 articles in 2024, representing a 162% growth [3]. Thermal energy storage technologies are classified into sensible [4], latent [5], and thermochemical heat storage [6].

Sensible heat storage relies on an increase in the temperature of the storage material. The amount of heat accumulated depends on the properties of the material (specific heat) and the temperature difference between the charging and discharging phases. Despite their simple design, these types of thermal energy storage systems are characterized by low heat storage density and limited thermal efficiency [7,8]. Latent heat storage utilizes materials that undergo a phase change. Phase Change Materials (PCMs) are divided into low-temperature, medium-temperature, and high-temperature categories. Low-temperature PCMs [9,10] are widely used in civil engineering, in the production of body-temperature-stabilizing wearables, and in refrigeration, e.g., in the transport of food, medicines, or cosmetics that require preserving low temperatures. Medium- and high-temperature PCMs [11] are used in waste heat recovery processes [12] and in power generation systems, such as Concentrated Solar Power (CSP) plants [13].

The main drawback of the aforementioned heat storage technologies results from thermal loss that limit the storage duration. Thermochemical Energy Storage (TCES) systems are free from this drawback, as they rely on a reversible chemical reaction or physical interactions thereby enabling long-term storage and high heat storage density [14]. In these systems, the heat storage density is about 8–10 times higher than for sensible heat storage and twice as high as for latent heat storage [15]. Research on heat storage technology using sorption focuses on the synthesis and characterization of new sorbent materials, the development of operation cycles, and the design of the storage units [16]. Although this technology is still in the laboratory research phase, attempts are being made to develop storage units in pilot scale [17]. Currently, commercial sorption heat storage systems are not available [18,19].

**Figure 1:**
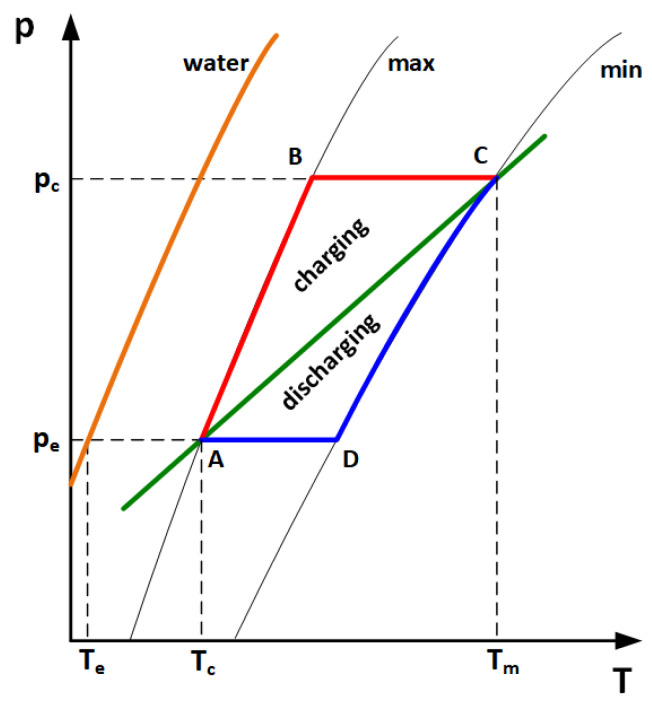
Thermodynamic cycle of zeolite heat storage units.

The adsorbent-water sorption pair is frequently studied for economic reasons [20]. Storage systems based on these materials operate in closed cycles. When heat is supplied to the system, the solid sorbent releases the working fluid, which must be condensed and stored in a separate vessel. The supply and adsorption of the working fluid by the solid sorbent can provide heat to the user due to the exothermic adsorption reaction [19]. With this operating cycle, these storages may serve as long-term storage solutions. Additionally, this technology also allows for high-density short-term storage [21,22]. Figure 1 shows a thermodynamic cycle of a zeolite heat storage unit.

The operation principle of a sorption heat storage unit involves four stages: charging and discharging. The charging stage includes two phases: isosteric heating (line A–B) and isobaric desorption (line B–C). The discharging stage is composed of an isosteric cooling (line C–D), and isobaric adsorption (line D–A). T_e_ and T_c_ represent the temperatures of the evaporator and condenser. The minimum and maximum temperatures of the cycle are determined by T_c_ and T_m_, respectively. Based on T_e_ and T_c_, it is possible to determine the values of the evaporator pressure p_e_ and condenser pressure p_c_.

In the group of ceramic materials, zeolites are materials with exceptional sorption properties [23], and zeolite 13X is of particular interest among them. It is characterized by a high capacity for water adsorption even at low pressure, which makes it an ideal material for adsorption cooling systems and thermochemical heat storage [24]. Its high adsorption capacity at temperatures up to 100 °C further emphasizes its effectiveness [25].

Zeolite 13X is also characterized by a low cost (2–3 € per kg), commercial availability, as well as high heat of adsorption and mechanical stability [26,27]. Research on this material focuses on both experimental analyses and numerical modeling of water vapor adsorption processes [28]. The developed numerical models allow simulation of thermodynamic processes occurring in adsorption reactors, which forms the basis for further optimization of thermochemical heat storage systems. Studies focus on detailed analysis at the laboratory scale [29,30,31] and demonstrate that tuning the input parameters of a heat storage system utilizing Zeolite 13X, such as the velocity and temperature of the working fluid at the inlet to the tested volume, significantly impacts the adsorption process dynamics [32].

Research focused on the use of zeolite 13X in heat storage systems places particular emphasis on systems where the heat transfer fluid is simultaneously the adsorbate, meaning it has direct contact with the adsorption material. The key objective is optimization of the adsorption and desorption process, which allows for effective storage and subsequent recovery of thermal energy.

One significant aspect analyzed in the studies is the influence of the temperature and humidity of the charging air on heat storage effectiveness [33,34]. It has been shown that a higher charging temperature leads to an increase in the energy density stored in the zeolite, whereas an increase in relative air humidity lowers the system’s Coefficient of Performance (COP). This implies that optimal system operating conditions require not only providing a sufficiently high temperature but also controlling the air humidity level, which is crucial for the efficiency of the entire process [35].

The research also addressed the issue of zeolite usage uniformity. It was demonstrated that the non-uniform utilization of the adsorption material can lead to a reduction in reaction efficiency, indicating the need for a more precise analysis of the temperature distribution and adsorption kinetics within different zones of the bed. Numerical models were developed, and experiments on beds of various shapes and sizes were conducted to better understand the mechanisms governing this process.

Another important issue raised in the studies is the possibility of simultaneously utilizing different energy sources for adsorbent regeneration [20]. Tested combinations included solar energy with low-temperature waste heat, which allows for more sustainable and economical system operation. The results indicate that this approach can improve the efficiency of adsorption and regeneration, thereby expanding the practical applicability of zeolite heat stores [36].

The research also tackled the issue of system scaling—investigating whether small-scale systems can reproduce the behavior of large-scale systems [31]. Experimental results suggest that in small reactors it is possible to achieve similar thermal output values in a shorter time, but doubts arise regarding the application of the same principles to significantly larger systems. Particularly important here are the differences resulting from the reactor geometry and the time scale of the adsorption and desorption processes.

The studies summarized in Table 1 involved both laboratory experiments and tests on larger prototype systems to evaluate the impact of various operational and design parameters on system performance.

During the discharging of a heat storage unit filled with a sorption material, the exothermic adsorption process of water or steam is utilized, whereas the endothermic reverse process (desorption) occurs during the charging of the unit. The discharging process presents a greater challenge than charging, because the heat flux recovered by the working fluid is conditioned by both the adsorption kinetics and the efficiency of heat exchange between the zeolite bed, the heat exchange system, and the working fluid. This is particularly significant in the case of systems that feature a heat exchange system, i.e., where the adsorbate and the working fluid are separated.

Numerous studies show that the transport of water vapor into the interior of the zeolite porous structure is the limiting step due to insufficient adsorption kinetics and diffusion resistance within the material [7,28,51]. Furthermore, local temperature increase in the zeolite bed during adsorption is thermodynamically unfavorable and results in a reduction in the adsorption kinetics. The authors [51] demonstrated that the adsorption rate can be up to 50% lower than the desorption rate under the same conditions. This phenomenon is widely described in numerous studies [52,53,54]. The aforementioned factors limit the achievable discharging power.

Research available in the literature [55] concerning the application of adsorption and desorption processes for heat storage focuses on analyzing phenomena at a relatively small scale, using heat storage models with a simplified design. This typically means that heat supply and heat extraction are achieved by the flow of the heat transfer fluid directly through the sorption bed, along with the adsorbed substance. This solution does not correspond to the design of actual heat storage units, where the heat transfer fluid is separated from the adsorbent bed and the adsorbate. However, the separation of the heat transfer fluid from the bed and the adsorbate leads to a reduction in the heat transfer intensity, and consequently, lower thermal power and lower efficiency.

In sorption storage systems, the efficiency of the charging and discharging processes depends on two types of losses: internal and external. External losses primarily result from the effectiveness of the thermal insulation, whereas internal losses result from limitations in mass transfer within the sorption bed and its porous structure, the uniformity of supplying the adsorbate to the sorption bed volume, and the actual adsorption capacity. While limiting external losses is possible by applying more effective thermal insulation, limiting internal losses poses a greater challenge. Internal efficiency is affected by the following factors: the actual adsorption capacity of the sorbent, the degree of utilization of the sorption bed, uniform distribution, and the selection of the optimal adsorption fluid flow rate. Furthermore, the heat transfer between the sorption bed and the heat transfer fluid is significant, as it occurs across several thermal barriers, such as the porous sorbent bed or the wall and finning of the tube through which the fluid flows.

Research on sorption storages of aforementioned design, particularly at a larger scale, is very limited and usually does not include a comprehensive analysis of factors affecting efficiency. This article aims to fill a gap in knowledge in this area.

The objective of the presented research was to determine the dynamic parameters during the discharging process of a sorption heat store equipped with a separate heat transfer fluid flow system. An important part of the analysis conducted was the heat balance calculated under dynamic conditions, considering both ambient heat losses (external) and internal losses. Based on this, two efficiencies were determined: the efficiency of the sorption bed and the total efficiency of the heat storage unit.

## 2. Materials and Methods

### 2.1. Laboratory Setup

**Figure 2:**
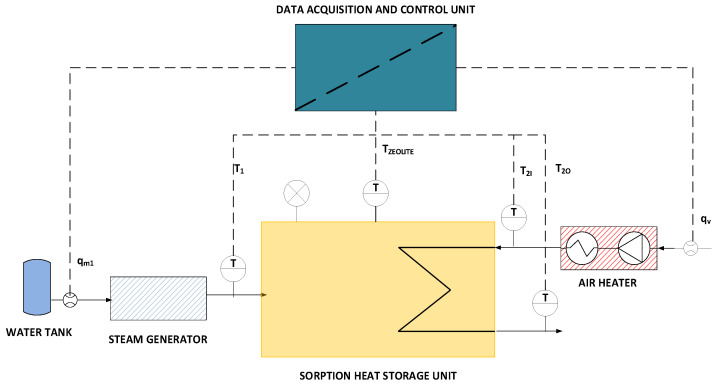
Scheme of the laboratory stand used in the study.

The scheme of the laboratory stand is presented in Figure 2. It is composed of a sorption heat storage unit filled with zeolite, an air heater, a steam generator, a data acquisition and control unit, as well as temperature and flow sensors. The list of components and their key parameters is shown in Table 2.

**Figure 3:**
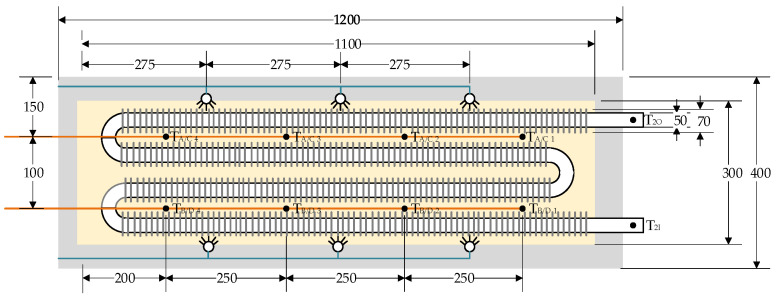
Internal design of the heat storage unit.

The internal design of the heat storage unit is presented in Figure 3. The essential feature of the presented heat storage unit is the separation of air flow from the zeolite bed to avoid mixing of discharging air with steam. This also allows avoiding the negative impact of air impurities on the zeolite bed. This is achieved by a finned air pipe, immersed inside the zeolite bed. The internal diameter and wall thickness are equal to 50 mm and 5 mm wall thickness, respectively. The fin diameter and packing density are 80 mm and 200 fins per meter, respectively. The role of the fins and multiple U-shaped pipes is to increase the heat transfer surface between the tube surface and the zeolite bed. Uniform injection of steam into the zeolite bed, is performed by six nozzles, located three on each side.

**Figure 4:**
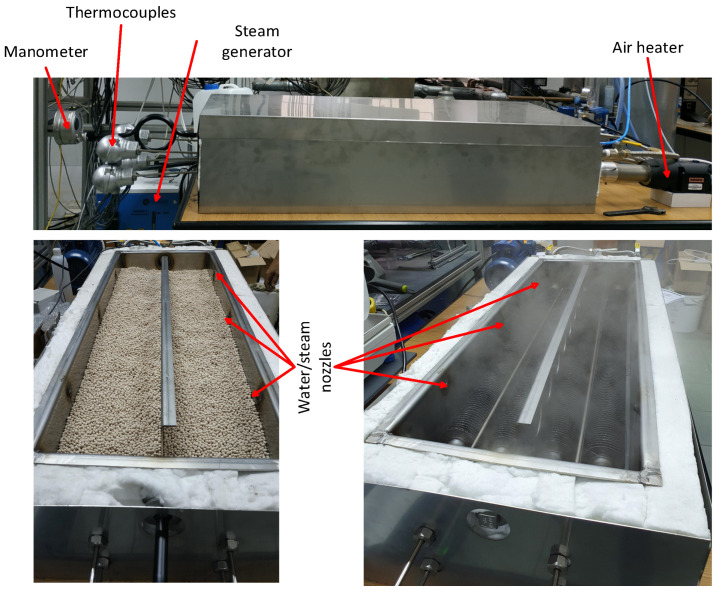
View of the heat storage unit: closed casing (**upper figure**), internal tray filled with zeolite (**bottom left**), preliminary test of steam nozzles (**bottom right**).

The zeolite bed and the finned tube are placed in a 1100 mm × 300 mm × 180 mm stainless steel tray, which is equipped with 50 mm-thick thermal insulation with λ = 0.031 W/m·K. The insulated tray is enclosed in a 1200 mm × 400 mm × 280 mm stainless steel casing (Figure 4).

### 2.2. Operation and Control of the Laboratory Stand

**Figure 5:**
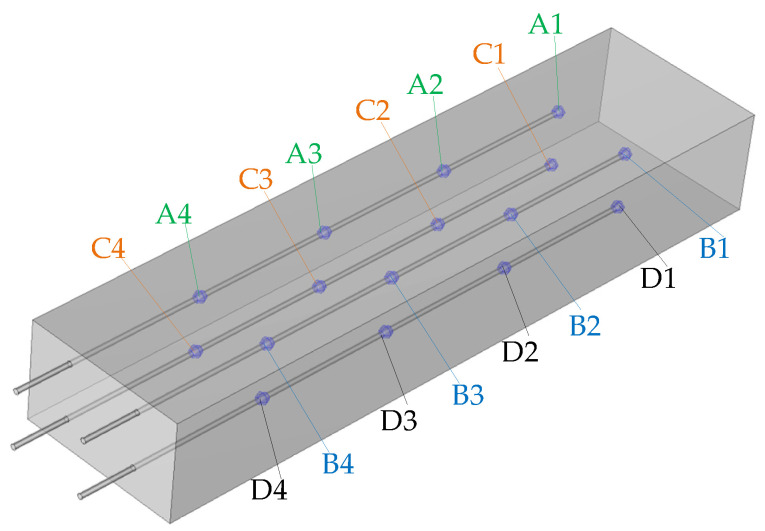
Location of temperature measurement points inside the zeolite bed.

During the discharging process, air at a constant temperature is supplied to the pipe using the air heater configured to its minimum temperature output. Simultaneously, steam is injected into the zeolite bed through six nozzles to initiate the exothermic adsorption reaction. As a result, heat generated during the adsorption reaction is transferred from the zeolite bed through the pipe walls to the air stream, which elevates the air temperature. Air and zeolite temperatures are measured using two single-point and four multipoint type J thermocouples. The thermocouples used within the assumed temperature range operate with an accuracy of ±2.5 °C (or 0.0075 T). Multipoint thermocouples have four sensors located at 1100 mm, 850 mm, 600 mm, and 350 mm along their length. Two single-point thermocouples are located at the air inlet and outlet. The remaining four were positioned within the zeolite bed (Figure 5). Each of the thermocouples placed within the bed possesses four measuring points distributed along its length: at 1100 mm, 850 mm, 600 mm, and 350 mm, which were designated as 1, 2, 3, and 4, respectively.

For system control, a PID controller with a neural network prediction layer was employed, similar to the pilot-scale phase-change heat storage unit investigated by the Authors in one of their previous publications [56].

The applied control system was responsible for adjusting the setpoint of air temperature and flow, as well as steam flow metering. While air control is a relatively simple task, optimization of steam flow is a more complex operation. It must be emphasized that the discharging power of a sorption heat storage unit varies depending on the heat flux generated by the adsorption reaction, zeolite bed temperature, reaction rate, and zeolite bed saturation. To preserve constant discharging power, the zeolite bed temperature and steam flow rate must be adjusted with respect to zeolite bed saturation. Furthermore, there is significant latency between a change in steam flow rate and the zeolite bed response. This issue is visible in the results published by Gao et al. [43], where a relatively small system was studied, as well as in the pilot-scale system investigated by Gabbrielli et al. [57]. In both cases, the stability of heat flux during the discharging process shows significant potential for improvement.

For this reason, a classical PID controller is insufficient for effective stabilization of the discharging power. The role of the employed neural network predictor was to dynamically optimize the steam flow rate required for stable discharging power, based on zeolite bed temperature and zeolite saturation level. The zeolite saturation level was estimated based on the total mass of steam introduced into the zeolite bed and the total adsorption capacity of the zeolite.

### 2.3. Experimental Methodology

The discharging process was performed using the laboratory stand described in Section 2.1. Air, as the heat transfer fluid, was supplied at a mass flow rate of 14.4 kg/h and a temperature of 40 °C (Table 3). Subsequently, the adsorption reaction was initiated by injecting steam into the zeolite bed at an average mass flow rate of 1.04 kg/h, which was then regulated by the control system. During the entire experiment, the parameters required for the balance calculations were measured, i.e., air inlet and outlet temperatures, air mass flow rate, and zeolite bed temperature distribution.

### 2.4. Heat Balance Model

The aim of the research was to perform complete balance calculations, which included the following components: heat flux generated by the adsorption reaction in the zeolite bed, heat flux to the discharging air stream, heat supplied by steam, and heat flux resulting from external heat loss. The aforementioned values were compared to the measured adsorption heat on 13X zeolite.

**Figure 6:**
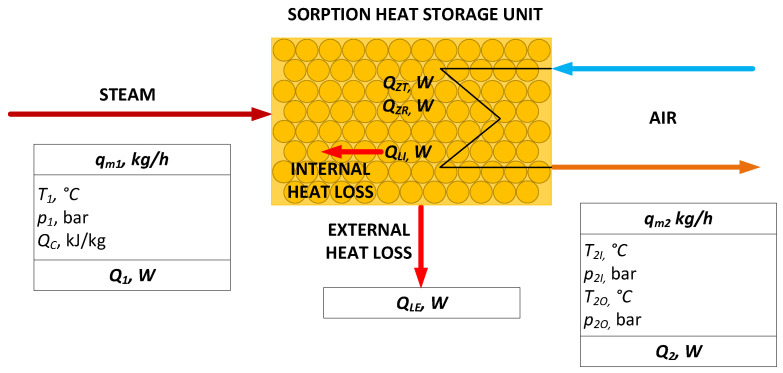
Balance scheme of the process, q_m1_—steam mass flow rate, T_1_—temperature of inlet steam, p_1_—pressure of inlet steam, Q_c_—heat of steam condensation, Q_1_—heat flux supplied by steam including condensation in the zeolite bed, Q_ZR_—real heat flux generated by zeolite-water adsorption, Q_ZT_—theoretical heat flux generated by zeolite-water adsorption, Q_LI_—internal heat loss, Q_LE_—external heat loss, q_m2_—air mass flow rate, T_2I_—inlet air temperature, p_2I_—inlet air pressure, T_2O_—outlet air temperature, p_2O_—outlet air pressure, and Q_2_—heat flux discharged by the air.

The scheme of the heat balance model is presented in Figure 6.

Governing equation of the heat balance is represented by Equation (1)

(1) $$Q_{1}+Q_{ZR}+Q_{LE}+Q_{2}=0.$$

Internal heat loss (*Q_LI_*) is the difference between the theoretical heat flux generated by zeolite-water adsorption, determined from zeolite properties measurements (*Q_ZT_*) and real heat flux generated by zeolite-water adsorption (*Q_ZR_*). This relation may be introduced into Equation (1), resulting with Equation (2)

(2) $$Q_{1}+\left(Q_{ZT}-Q_{LI}\right)+Q_{LE}+Q_{2}=0.$$

Heat flux supplied by steam is calculated using Equation (3)

(3) $$Q_{1}=q_{m1}\left(H_{1I}-H_{1B}\right),$$

where

*H*_1*I*_—enthalpy of inlet steam,

*H*_1*B*_—enthalpy of steam at a temperature equal to average zeolite bed temperature.

Enthalpy values were determined from CoolProp library [58].

Analogously, heat flux discharged by air stream is expressed by Equation (3)

(4) $$Q_{2}=q_{m2}\left(H_{2I}-H_{2O}\right),$$

where

*q_m_*_2_—air mass flow rate,

*H*_2*I*_—enthalpy of inlet air,

*H*_2*O*_—enthalpy of outlet air.

In order to determine the external heat loss, the methodology from Schreiber et al. [50] was adopted, supplemented by appropriate equations from Churchill and Chu [59].

The external heat loss (*Q_LE_*) is calculated using the thermal resistance for a composite wall, composed of zeolite bed, tray wall, insulation and casing wall. The expression Equation (5) is based on the temperature difference across the wall and the total thermal resistance (*R_TOT_*)

(5) $$Q_{LE}=\frac{A\left(T_{Z}-T_{EXT}\right)}{R_{TOT}},$$

where

*A*—external surface area of the heat storage unit casing,

*T_Z_*—average zeolite temperature,

*T_EXT_*—external (ambient) temperature,

*R_TOT_*—total thermal resistance of the composite wall.

Total thermal resistance is the sum of the conductive resistances of the individual layers of the composite wall and the external convective resistance, Equation (6)

(6) $$R_{total}=\left(\frac{d_{zeo}}{\lambda_{zeo}}\right)+\left(\frac{d_{st1}}{\lambda_{st}}\right)+\left(\frac{d_{ins}}{\lambda_{ins}}\right)+\left(\frac{d_{st2}}{\lambda_{st}}\right)+\left(\frac{1}{\alpha_{ext}}\right),$$

where

$d_{zeo}$—zeolite thickness,

$d_{st1}$—first layer of casing thickness,

$d_{ins}$—insulation thickness,

$d_{st2}$—second later of casing thickness,

$\lambda_{zeo}$—zeolite thermal conductivity,

$\lambda_{st}$—steel thermal conductivity,

$\lambda_{ins}$—insulation thermal conductivity,

$\alpha_{ext}$—external convective heat transfer coefficient.

The final expression for the external heat loss is represented by Equation (7)

(7) $$Q=\frac{A\cdot \left(T_{Z}-T_{EXT}\right)}{\frac{d_{zeo}}{\lambda_{zeo}}+\frac{d_{st1}}{\lambda_{st}}+\frac{d_{ins}}{\lambda_{ins}}+\frac{d_{st2}}{\lambda_{st}}+\frac{1}{\alpha_{ext}}},$$

where

$d_{zeo}$—zeolite thickness,

$d_{st1}$—first layer of casing thickness,

$d_{ins}$—insulation thickness,

$d_{st2}$—second later of casing thickness,

$\lambda_{zeo}$—zeolite thermal conductivity,

$\lambda_{st}$—steel thermal conductivity,

$\lambda_{ins}$—insulation thermal conductivity,

$\alpha_{ext}$—external convective heat transfer coefficient.

The convective heat transfer coefficient is determined by natural convection, which is calculated using Equation (8):

(8) $$\alpha_{ext}=\frac{Nu\cdot \lambda_{air}}{L}$$

where

*λ_air_*—thermal conductivity of air,

*L*—characteristic length,

*Nu*—Nusselt number.

The Nusselt correlation used in the calculations is expressed by Equation (9)

(9) $$Nu={\left(0.825+0.387{\left(Gr\cdot Pr\cdot f_{1}\right)}^{1/6}\right)}^{2}+0.435\frac{L}{d_{ins}}$$

(10) $$f_{1}={\left(1+{\left(\frac{0.492}{Pr}\right)}^{\frac{9}{16}}\right)}^{-\frac{16}{9}}\;Gr=\frac{g\cdot \beta_{air}\left(T_{Z}-T_{EXT}\right)\cdot L^{3}}{{\nu_{air}}^{2}}\cdot$$

(11) $$Pr=\frac{\mu_{air}C_{p,\;air}}{\lambda_{air}}$$

where

*Pr*—Prandtl number,

*Gr*—Grashof number,

*β_air_*—thermal expansion coefficient of air,

*g*—gravity,

*C_p,air_*—heat capacity of air,

*μ_air_*—dynamic viscosity of air,

*ν_air_*—kinematic viscosity of air.

Two types of efficiency were determined: zeolite bed efficiency (Equation (12)) and total discharging efficiency (Equation (13)):

(12) $$\eta_{Z}=\frac{Q_{ZR}}{Q_{ZT}}\cdot 100\%$$

(13) $$\eta_{T}=\frac{Q_{2}}{Q_{ZT}}\cdot 100\%$$

Physical constants, properties and other input values are presented in Table 4.

## 3. Results and Discussion

**Figure 7:**
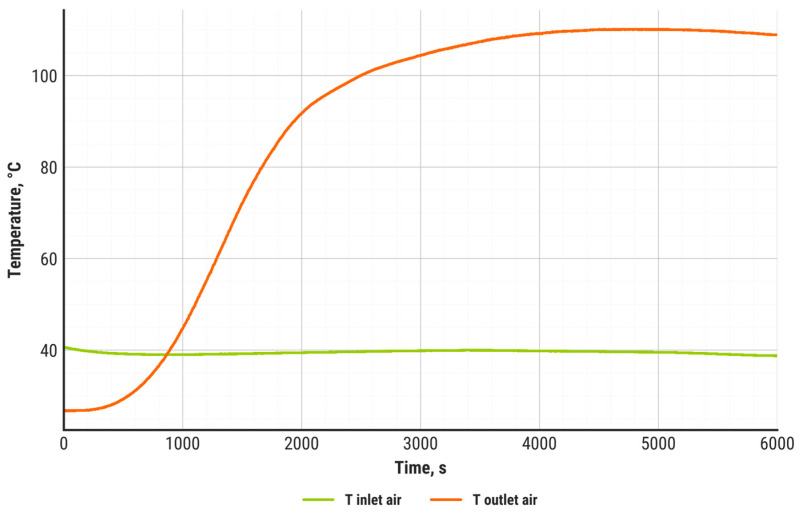
Inlet and outlet air temperature in time.

During the discharging process, the inlet air temperature (Figure 7, green) was maintained constant at 40 °C. Due to ambient conditions and the initial temperature of the heat storage unit, including the zeolite bed, the outlet air temperature was initially lower. During the first 2000 s of discharging, the system reached stability, and after 3000 s operated at steady state, when the outlet air temperature remained stable at around 110 °C. The results presented in this study demonstrate a substantial improvement compared to other published studies (Table 1), where the maximum outlet temperature reported was 70 °C.

**Figure 8:**
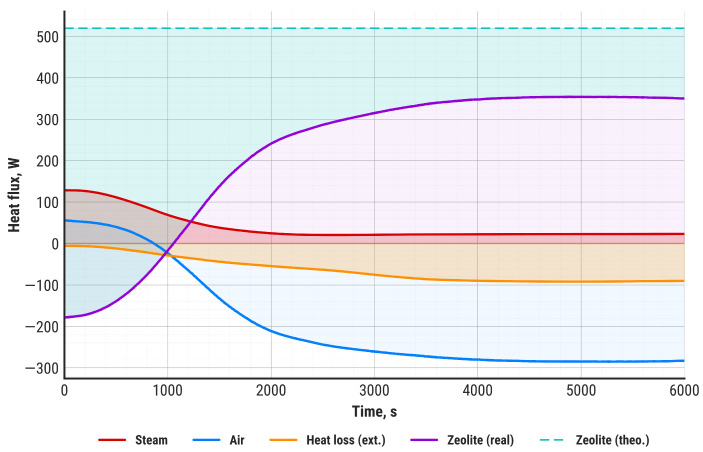
Heat fluxes referring to heat balance components.

Balance calculations were performed for each measurement point to obtain the dynamic balance of the heat storage unit during the entire experiment. For this purpose, the methodology presented in Section 2.4 was employed. The results are presented in Figure 8. The heat flux discharged by the air stream (Q_2_) is represented in blue, the heat flux supplied by steam (Q_1_) in red, the actual heat flux generated by the zeolite–water adsorption reaction (*Q_ZR_*) in violet, and the heat flux resulting from external thermal loss (*Q_LE_*) in orange. Additionally, the theoretical heat flux, calculated using the zeolite–water heat of adsorption, is indicated with a green dashed line. Similarly to the air temperature profile, the analyzed heat fluxes reach stability after 2000 s of the discharging process, while after 3000 s, the system operates at steady state.

It must be emphasized that both the outlet air temperature and the net discharging power remain stable until the end of the experiment. Compared to the results obtained in other studies, e.g., by Gao et al. [43] or Gabbrielli et al. [57], our system demonstrated a significant improvement.

**Figure 9:**
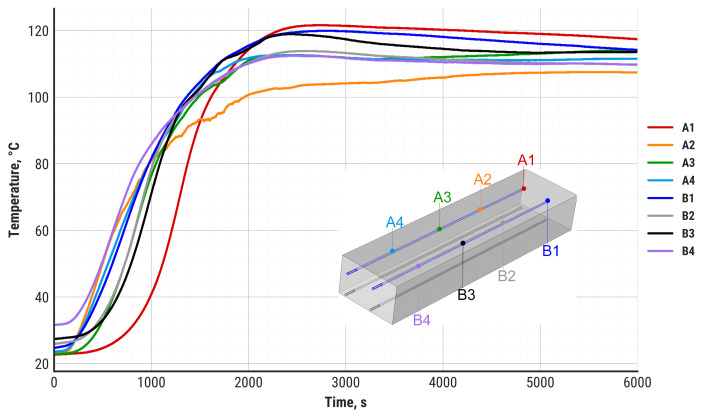
Temperature of the zeolite in the upper layer of the bed during the experiment.

**Figure 10:**
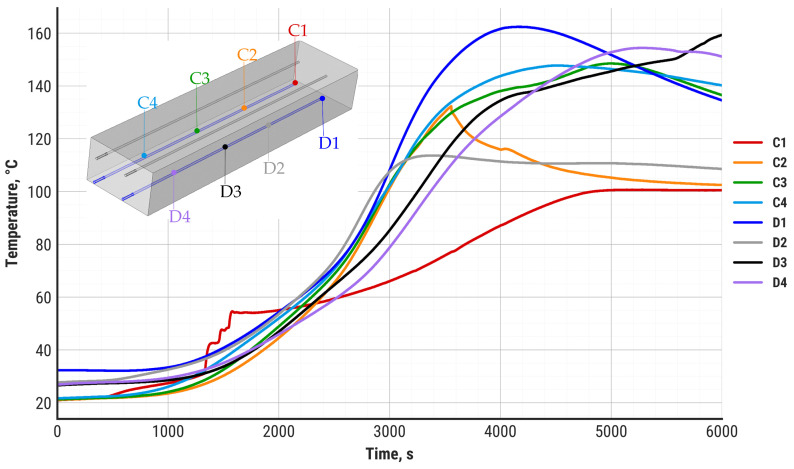
Temperature of the zeolite in the bottom layer of the bed during the experiment.

During the study, the temperature distribution in the zeolite bed was also recorded. The results are divided into the top-layer temperature distribution (A and B sensors, Figure 9) and the bottom-layer temperature distribution (C and D sensors, Figure 10).

The upper layers of the zeolite bed reach a maximum temperature of 100–120 °C after approximately 2000 s, which corresponds to the heat flux and air temperature stabilization presented in Figure 8 and Figure 9. The bottom layers demonstrate a substantial delay in temperature increase and reach maximum values between 3000 s and 4000 s of discharging. There are two main differences visible between the zeolite top layers and bottom layers: a substantially larger temperature gradient in the bottom layers and a higher maximum temperature value. While the top layers of the zeolite bed do not exceed 120 °C, some of the bottom layers can reach up to 162 °C at the maximum point. It is worth noting that the zeolite bed temperature around the air inlet zone (C1, C2 sensors) stabilizes at 100 °C and remains at this level until the termination of the discharging process. This effect is probably the result of steam condensation in the zeolite bed around the air inlet zone.

Detailed steady-state balance calculations, based on the data between 4000 s and 6000 s, are presented in Table 5.

**Figure 11:**
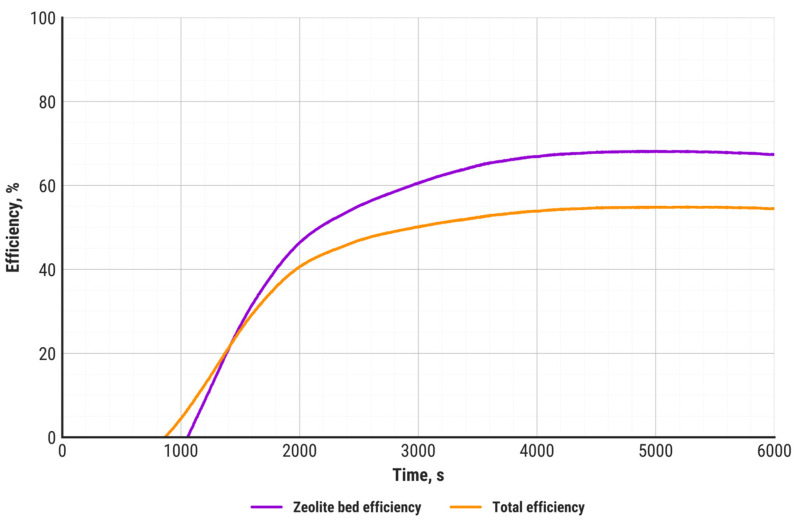
Sorption bed efficiency and total efficiency during heat storage unit discharging.

Zeolite bed efficiency (violet curve) and total efficiency (orange) during the entire discharging process are presented in Figure 11.

**Figure 12:**
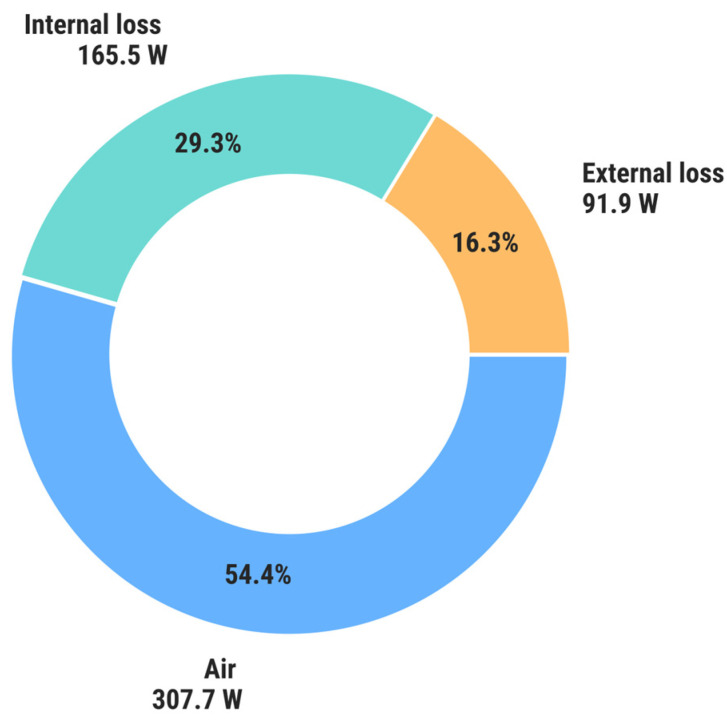
Heat flux distribution between discharging air (blue), internal loss (green) and external loss (orange), in steady state.

Figure 12 shows the zeolite heat flux distribution between discharging air (blue), internal loss (green) and external loss (orange), in steady state.

The total efficiency of the discharging process, referred to the theoretical zeolite–water adsorption reaction heat, is 54.4%, which includes both internal and external heat losses. Excluding external losses, the zeolite bed efficiency reaches 70%. The zeolite efficiency depends on reaction kinetics, mass transfer of water molecules inside the microporous structure of the zeolite, as well as the uniformity of steam injection within the zeolite bed volume. The last of these factors can be identified by the temperature distribution inside the zeolite. In this study, it was evidenced that, except for the air inlet zone, the zeolite temperature distribution was uniform, which means that the applied steam injection method was optimal. It must be emphasized that the zeolite bed efficiency is based on the adsorption capacity determined using the DSC-TGA technique, with the assumption that the adsorption and desorption capacities are equal. The actual adsorption capacity may be reduced depending on the temperature, pressure, and diffusion of water molecules inside the zeolite’s porous structure. Identification of the impact of the aforementioned factors requires an extensive investigation of zeolite properties.

## 4. Uncertainty Analysis

Based on the accuracy of the measuring instruments and the assumed physical constants, the uncertainty in determining the zeolite bed efficiency ${∆\eta }_{Z}$ and the total discharge efficiency $∆\eta_{T}$ was calculated. The total differential method was applied to determine the uncertainty, which is represented by the following equations:

(14) $$∆\eta_{Z}=\frac{\partial }{\partial Q_{ZR}}\frac{Q_{2}}{Q_{ZT}}∆Q_{2}+\frac{\partial }{\partial Q_{ZT}}\frac{Q_{2}}{Q_{ZT}}∆Q_{ZT}$$

(15) $$∆\eta_{T}=\frac{\partial }{\partial Q_{2}}\frac{Q_{2}}{Q_{ZT}}∆Q_{ZR}+\frac{\partial }{\partial Q_{ZT}}\frac{Q_{ZR}}{Q_{ZT}}∆Q_{ZR}$$

The maximum values of the relative and absolute errors of the measured and determined quantities are given in Table 6. The zeolite bed efficiency was determined with a relative error smaller than ±3.98%.

## 5. Conclusions

The aim of the experimental study was to investigate the dynamics of the steam adsorption process on zeolite 13X and to determine the total system efficiency and the zeolite bed efficiency. The designed system enabled the separation of air flow from the zeolite bed, which prevented direct contact of steam with the heat transfer fluid and contamination of the bed. A PID controller with a predictive layer based on a neural network was used to control the process, ensuring stable discharge power. A balance model was developed to determine the total system efficiency and the zeolite bed efficiency. In the steady state, a heat flux transferred to the air of approximately 300 W was obtained, which allowed the air to be heated from 40 °C to 110 °C. This value is significantly higher than those reported by other researchers. The total efficiency of the discharge process, considering internal and external losses, was 54.4%, while the zeolite bed efficiency reached 70%. The results confirmed the high effectiveness of the developed system and its advantage over other solutions described in the literature. The element of novelty in the conducted research is the higher operating stability of the sorption heat storage achieved by using a heat exchange system that separates the working fluid from the adsorbate and by employing a neural network-based control layer for predicting the steam mass flow rate based on temperature measurements in the zeolite bed and the zeolite saturation, estimated from the total mass of steam introduced into the bed and the adsorption capacity of the zeolite.

A comprehensive comparison of the charging and discharging processes using the neural network-based control model is planned for publication as a separate article in the future.

Key Conclusions from the sorption thermal energy storage research:

1. The use of a control layer employing neural networks to optimize the steam flow rate improves the operational stability of the sorption thermal energy storage system.
2. The obtained total efficiency of the heat storage system and the zeolite bed efficiency are comparable to those reported by other researchers. The total storage efficiency was approximately 55%, while the zeolite bed efficiency was around 70%.

## Figures and Tables

**Table 1:** Comparison of thermochemical heat storage systems at laboratory scale.

Authors	Storage Shape	Storage Dimensions	Zeolite Amount	Adsorbate Temperature During Delivery	Maximum Temperature at the Outlet of the Working Medium	Heat Exchange System
[37]	column	H: 350 mm D: 30 mm	H: 200 mm D: 30 mm	-	-	No
[38]	box	H: 126 mm L: 268 mm W: 221 mm	35 × 268 × 221 mm	21 °C	37 °C	No
[39]	box	b.d.	90 mm × 90 mm × 50 mm	-	-	No
[40,41]	cylinders	4.16 × 2.2 × 1.6 m	80 kg	20	57	No
[31,42]	column	H: 100 mm D: 44 mm	H: 100 mm D: 38 mm	22	53	No
[31,43]	column	H: 168 mm D: 86 mm	H: 120 mm D: 76 mm	18	37	No
[44]	column	H: 1673 mm D:159.3 mm	H: 300 mm D:159.3 mm	-	-	No
[45]	cylinder	H: 300 mm D: 700 mm	45.15 kg	25	60	No
[46]	box	1000 mm × 300 mm × 33 mm	41 kg	20	54	No
[47,48]	box	500 mm × 459 mm × 500 mm	62.5 dm3	13	35	No
[49]	box	-	200 dm3	17	36	Yes
[50]	column	-	10 kg	40	70	Yes

**Table 2:** Selected parameters of the laboratory setup components.

Component	Main Parameters
Air heater: Leister Mistral 6 (Leister, Kaegiswil, Switzerland)	6–21 m^3^/h (20 °C), 40–500 °C
Steam generator: Batistella Barbara 31 (Batistella, Rossano, Italy)	1450 W, 2.8 bar
Air flowmeter: MV 106 (Mass Flow Online, Bronkhorst, The Netherlands)	Max 500 L/min, accuracy: ±(1% RD + 0.5% FS)
Thermocouples type J (Termoaparatura Wrocław, Wrocław, Poland)	−40–750 °C, 55 µV/°C.

**Table 3:** Experiment parameters.

Parameter	Value
Air mass flow rate, kg/h	14.4
Inlet air temperature, °C	40
Zeolite bed mass, g	2270
Average steam flow rate, kg/h	1.04

**Table 4:** Physical constants, physical properties and other input values.

Parameter	Value
Air mass flow rate, kg/h	14.4
Inlet air temperature, °C	40
Zeolite bed mass, g	2270
Average steam flow rate, kg/h	1.04
Zeolite thickness, mm	100
Casing (steel) thickness, mm	5
Insulation thickness, m	50
Characteristic dimension of casing, mm	300
Zeolite thermal conductivity, W/(m∙K)	0.07
Insulation thermal conductivity, W/(m∙K)	0.07

**Table 5:** Balance calculations results in steady state.

	Parameter	Value
Steam	q_m1_, kg/h	1.12
	T_1_, °C	130
	p_1_, bar	2.8
	Q_1_, W	21.6
TES	Q_ZT_, W	565.1
	Q_ZR_, W	399.6
	Q_LI_, W	165.5
	Q_LE_, W	91.9
Air	q_m2_, kg/h	14.4
	T_2I_, °C	40
	T_2O_, °C	2
	Q_2_, W	307.7
	η_T_, %	54.4
	η_Z_, %	70.1

**Table 6:** The accuracy of the measuring instruments calculeted the uncertainty in determining the zeolite bed efficiency and the total discharge efficiency.

Parameter	Value
Viscosity, heat capacity, thermal conductivity, thermal expansion coefficient	±1%
Mass flow rate	±1%
Temperature	±2 °C
Dimensions	±1 mm
Zeolite bed efficiency	±3.98%
Total discharge efficiency	±1.00%

## Data Availability

The original contributions presented in this study are included in the article. Further inquiries can be directed to the corresponding author.
